# Functional Importance of the DNA Binding Activity of *Candida albicans* Czf1p

**DOI:** 10.1371/journal.pone.0039624

**Published:** 2012-06-27

**Authors:** Ivana Petrovska, Carol A. Kumamoto

**Affiliations:** 1 Graduate Program in Genetics, Sackler School of Graduate Biomedical Sciences, Tufts University, Boston, Massachusetts, United States of America; 2 Department of Molecular Biology and Microbiology, School of Medicine, Tufts University, Boston, Massachusetts, United States of America; New Jersey Medical School, University of Medicine and Dentistry of New Jersey, United States of America

## Abstract

The human opportunistic pathogen *Candida albicans* undergoes a reversible morphological transition between the yeast and hyphal states in response to a variety of signals. One such environmental trigger is growth within a semisolid matrix such as agar medium. This growth condition is of interest because it may mimic the growth of *C. albicans* in contact with host tissue during infection. During growth within a semisolid matrix, hyphal growth is positively regulated by the transcriptional regulator Czf1p and negatively by a second key transcriptional regulator, Efg1p. Genetic studies indicate that Czf1p, a member of the zinc-cluster family of transcriptional regulators, exerts its function by opposing the inhibitory influence of Efg1p on matrix-induced filamentous growth. We examined the importance of the two known activities of Czf1p, DNA-binding and interaction with Efg1p. We found that the two activities were separable by mutation allowing us to demonstrate that the DNA-binding activity of Czf1p was essential for its role as a positive regulator of morphogenesis. Surprisingly, however, interactions with Efg1p appeared to be largely dispensable. Our studies provide the first evidence of a key role for the DNA-binding activity of Czf1p in the morphological yeast-to-hyphal transition triggered by matrix-embedded growth.

## Introduction

*Candida albicans* is a diploid fungus that has adapted primarily for survival as a commensal of mammalian hosts. It is a common member of the human microbiota of the gastrointestinal tract, mucus membranes and the skin. In instances when the host immune system is weakened (for example, during immunosuppression therapy or in neonates) or the competing microflora is eliminated (for example, during antibiotic treatment) the organism can cause a range of diseases [1]. Oral thrush is an example of a superficial mucosal infection caused by *C. albicans*. If the organism gains access to the bloodstream, a condition known as candidaemia, it may cause a life-threatening systemic disease. Such *Candida* infections are the fourth-leading cause of nosocomial disease in the United States [2].

The organism's adaptability for survival in a range of niches within the host is in great part due to its highly plastic behavior. For example, growth of *C. albicans* can be in the yeast form or either of two filamentous states: pseudohyphae or true hyphae [3]. The transition between the different morphological forms is reversible. Filamentous growth is thought to help *C. albicans* invade and penetrate tissue, escaping the original site of infection and reaching distal sites, while growth in the yeast form may be better suited for dissemination in the bloodstream. As a result, polymorphic growth is considered to be an important virulence trait of *C. albicans* [4]. This is supported by studies showing that mutants locked in one morphological state or another are attenuated for virulence [5], [6]. A mutant of *hgc1*, a hypha specific cyclin, that is defective in filamentous growth but expresses several hypha-associated genes, is also attenuated for virulence in the mouse model of systemic infection [7].

A variety of signals trigger the yeast-to-hyphal transition. In a laboratory setting, several of these are growth at 37°C in the presence of serum, alkaline pH or a nitrogen limiting environment [3]. A large network of connections is involved in the control of morphogenesis and is responsive to a range of hyphal-inducing signals. Two highly conserved pathways, the cAMP dependent protein kinase A (PKA) and the mitogen activated protein kinase (MAPK) pathway, are at the heart of the network and have a positive effect on the morphological switch [5]. Mutations in components that lie within the two pathways (for recent review, see [8]) and in particular, the downstream transcriptional regulators Efg1p of the cAMP-PKA pathway and Cph1p of the MAPK pathway, lead to defects in filamentous growth [5], [9], [10], [11]. A strain lacking both *EFG1* and *CPH1* exhibits a dramatic defect in filament formation during most hyphal-inducing conditions, and is avirulent in a model of systemic infection in the mouse [5]. However, interestingly, in an alternative model of *C. albicans* infection, an *efg1*Δ *cph1*Δ mutant is able to filament and invade tissue [12].

Growth within semi-solid agar matrix is an environmental condition that triggers morphogenesis, and may mimic the encounter and growth of *C. albicans* in contact with tissue [13]. Here, a control mechanism distinct from that seen during other hypha-inducing conditions at 37°C is active. Efg1p, required for filamentous growth at 37°C in a range of conditions, has a negative effect on this process during matrix-embedded growth at 25°C [14]. A second pathway mediated by the transcriptional regulator Czf1p relieves the inhibitory effect of Efg1p and promotes morphogenesis [13], [14]. Cph1p promotes filamentation, but has a more minor role during this process [14]. Ectopic expression or deletion of *CZF1* do not enhance or reduce filamentation during embedded growth in the absence of Efg1p, making the *efg1*Δ mutant phenotype epistatic to that of a *czf1*Δ mutant. The epistatic interactions during matrix-embedded growth at 25°C point to a functional relationship between Czf1p and Efg1p where Czf1p relieves Efg1p-mediated inhibition of filamentation.

*EFG1* encodes a DNA-binding protein of the APSES family of fungal transcriptional regulators with a multitude of functions in *C. albicans* [11], [15], [16], [17]. In the absence of *EFG1*, a number of genes are either up or downregulated [15], [16], [18], [19]. The precise timing and magnitude of *EFG1* expression is essential for its function in morphogenesis [11], [20]. Efg1p, characterized by a conserved helix-loop-helix (bHLH) domain involved in DNA-binding, binds to EGR elements found in promoter regions of target genes during yeast growth [21]. Induction of hyphal growth is associated with a shift of Efg1p binding away from these elements to several other sequences that have similarities with other regulatory sequences [21]. Efg1p is thought to direct downstream effects both directly and indirectly, through intermediate transcriptional regulators.

*CZF1* encodes a DNA-binding protein of the Cys_6_Zn_2_ class of transcriptional regulators [13], [22], [23]. *CZF1* expression is regulated by a variety of environmental conditions such as carbon source, growth phase, temperature and the physical environment, and the *CZF1* promoter region appears to be under complex regulatory control [17], [21], [24]. Interestingly, *CZF1* expression is Efg1p dependent under all conditions tested [24]. It is also subject to negative autoregulation [24]. A *czf1*Δ mutant has a defect in filamentation during embedded growth but not during other liquid based hypha-inducing conditions pointing to the specific role of Czf1p in the control of matrix-induced filamentation [13].

How Czf1p and Efg1p work together to control morphogenesis is not understood. Czf1p binds to DNA [23] and physically interacts with Efg1p [14]. The biological function of the protein's DNA-binding activity and the significance of the Czf1p-Efg1p interaction in the control of matrix-induced filamentation have not been previously studied. Here, we show that the DNA-binding activity of Czf1p and the ability to interact with Efg1p could be separated by mutation. We also show that DNA-binding of Czf1p is essential for its regulatory function and role as a positive regulator of filamentation, whereas interaction with Efg1p is dispensable for this function.

## Results

### *czf1* point mutants defective in the yeast-to-hyphal morphological transition during matrix-embedded growth

Czf1p belongs to a class of transcriptional regulators that possess a well-conserved Cys_6_Zn_2_ motif, with six cysteines binding to two zinc atoms and coordinating the fold of the domain [22]. While the majority of zinc-finger proteins bind to DNA, functionally they are very diverse. For example, the Cys_6_Zn_2_ motif has been implicated in physiological roles such as zinc sensing, chromatin remodeling and protein interactions [25], [26]. Whether the Cys_6_Zn_2_ motif of Czf1p is important for its role as a positive regulator of matrix-induced filamentation and is involved in DNA-binding had not been explored. We examined the possible contribution of residues within this domain to the function of Czf1p as a positive regulator of morphogenesis during embedded growth.

**Figure 1:**
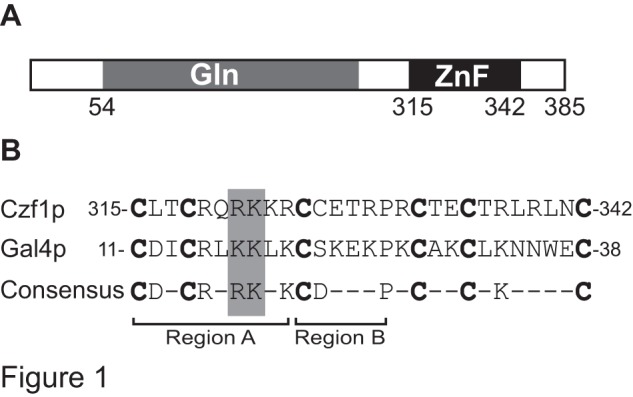
Conserved residues of Czf1p. (Panel A) Diagram of Czf1p. Numbers indicate amino acid residues. Gln, glutamine rich region. ZnF, zinc finger motif. (Panel B) Alignment of the zinc-finger motif amino acid sequence of Czf1p (residues 315–342) and *S. cerevisiae* Gal4p (residues 11–38). Marked regions A and B, and consensus sequence are from Corton et al. (1998) and are based on amino acids that are conserved in more than 33% of sequences from 79 Cys_6_Zn_2_ fungal proteins. Lys17 and Lys18 in Gal4p make direct contacts with the target DNA sequence. Underlined residues in the Czf1p amino acid sequence were targeted in this study.

Using the structure of *Saccharomyces cerevisiae* Gal4p, a DNA-binding protein and activator of *GAL* genes for comparison, we identified conserved residues in the putative zinc-finger domain of Czf1p that were predicted to contact DNA, Arg321 and Lys322 (FIG 1). In order to test their contribution to DNA-binding and Czf1p activity, we substituted each residue with an alanine. We also constructed an alanine substitution mutation of Thr328 because an analogous residue in Leu3p, His50, contributes to the function of the protein as a repressor [27].

Constructs carrying *CZF1-myc*, *czf1_T328A_-myc, czf1_R321A_-myc* or *czf1_K322A_-myc* were introduced into a *czf1*Δ *cph1*Δ null strain in order to test their effects on filamentation during matrix-embedded growth at 25°C. The double null mutant is very defective in filamentation under embedded conditions facilitating analysis of mutant phenotypes.

**Figure 2:**
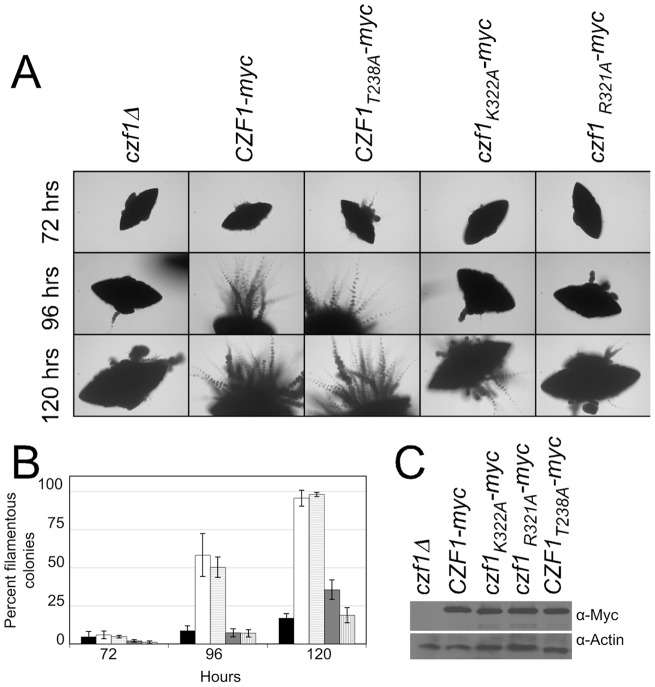
Filamentation of *czf1* mutants during growth under matrix-embedded conditions. Cells of *czf1*Δ *cph1*Δ mutant strains carrying Myc-tagged wild type *CZF1* under control of the maltase promoter, Myc-tagged mutant *CZF1* under control of the maltase promoter or vector alone were embedded in YPS + uridine, 1% agar and grown at 25°C. Two independent isolates of each strain were tested in duplicate. (Panel A) Representative colonies were photographed at 10x at the times indicated. (Panel B) The percentage of filamentous colonies for a given strain at various times was measured as described in Materials and Methods. Data show the mean of the 4 determinations and the standard deviation. Black bar, *czf1*Δ (IPC11); white bar, *CZF1* ^+^-myc (IPC22); horizontal lines, *CZF1*_T328A_-myc (IPC51); grey bar, *czf1*_K322A_-myc (IPC31); vertical lines, *czf1*_R321A_-myc (IPC42). (Panel C) Cells were grown in liquid YPS medium at 30°C and 30 µg of total protein was fractionated by SDS PAGE. Immunoblot analysis was performed using an anti-Myc antibody to detect tagged Czf1 proteins. Actin was detected with anti-actin as a loading control.

Strains carrying these *CZF1* alleles were grown embedded in YPS agar at 25°C and representative colony images are shown in Figure 2A. Filamentous colonies were counted at the times indicated, as described in Materials and Methods (FIG 2B). In strains carrying either wild-type *CZF1-myc* or *CZF1_T328A_-myc*, filamentation initiated at around the 72-hour time-point and about fifty percent of colonies were filamentous at 96 hours. Strains carrying *czf1_R321A_-myc* or *czf1_K322A_-myc* were highly defective in filamentation and resembled the *czf1*Δ *cph1*Δ double null mutant during the first 96 hours of growth under embedded conditions. After this time point, the *czf1_R321A_-myc* strain remained primarily non-filamentous like the *czf1*Δ *cph1*Δ double null strain, whereas the *czf1_K322A_-myc* strain showed some increase in filamentation. At 96 hours, differences in the percent of filamentous colonies between *CZF1-myc* and either *czf1_R321A_-myc* or *czf1_K322A_-myc* were statistically significant (Table 1).

**Table 1:** Defects in filamentation under embedded conditions at 96 hours.

Strain background	*CZF1* allele^a^	Percent filamentous colonies^b^	p value (vs *CZF1-myc*)^c^	p value (vs *CZF1* ^+^)^c^
*czf1*Δ *cph1*Δ Ura^−^	*czf1*Δ	9±3	<0.001	
*czf1*Δ *cph1*Δ Ura^−^	*CZF1-myc*	58±14		
*czf1*Δ *cph1*Δ Ura^−^	*CZF1*_T328A_-*myc*	50±7	<0.35	
*czf1*Δ *cph1*Δ Ura^−^	*czf1*_K322A_-*myc*	7±3	<0.001	
*czf1*Δ *cph1*Δ Ura^−^	*czf1*_R321A_-*myc*	7±2	<0.001	
*czf1*Δ *cph1*Δ Ura^+^	*czf1*Δ	4±1		<0.000001
*czf1*Δ *cph1*Δ Ura^+^	*CZF1* ^+^	98±3		
*czf1*Δ *cph1*Δ Ura^+^	*CZF1*_T328A_	98±3		<0.78
*czf1*Δ *cph1*Δ Ura^+^	*czf1*_K322A_	32±5		<0.0001
*czf1*Δ *cph1*Δ Ura^+^	*czf1*_R321A_	5±3		<0.00001

a Allele carried on plasmid, under control of Maltase promoter, integrated at *ADE2* locus.

b Mean±standard deviation; three determinations for untagged *CZF1* strains; four determinations for tagged *CZF1* strains.

C By two-tailed *t* test.

When untagged alleles were analyzed in a Ura^+^ strain background, similar relative effects were observed (Table 1), demonstrating a statistically significant defect in formation of filamentous colonies for *czf1_R321A_* and *czf1_K322A_* strains in comparison to the *CZF1^+^* strain. Furthermore, expression of a *czf1* mutant in a strain where both copies of *CZF1* and *CPH1* were present on the chromosome did not cause a defect in filamentation suggesting that the *czf1* mutants were loss of function mutants (data not shown).

To show that the defective mutant proteins were produced normally, extracts of cells carrying myc-tagged alleles were analyzed by immunoblotting analysis with anti-myc antibody. Results showed that wild-type and mutant Czf1 proteins were present at similar levels (FIG 2C).

These results demonstrate that, as in Gal4p and other members of this family, two residues in region A of Czf1p, the highly conserved Arg321 and Lys322, contribute to the function of the protein in invasive filamentation. In contrast, Thr328 in region B does not appear to be important.

### *czf1* mutants are defective in DNA-binding to a target DNA sequence

**Figure 3:**
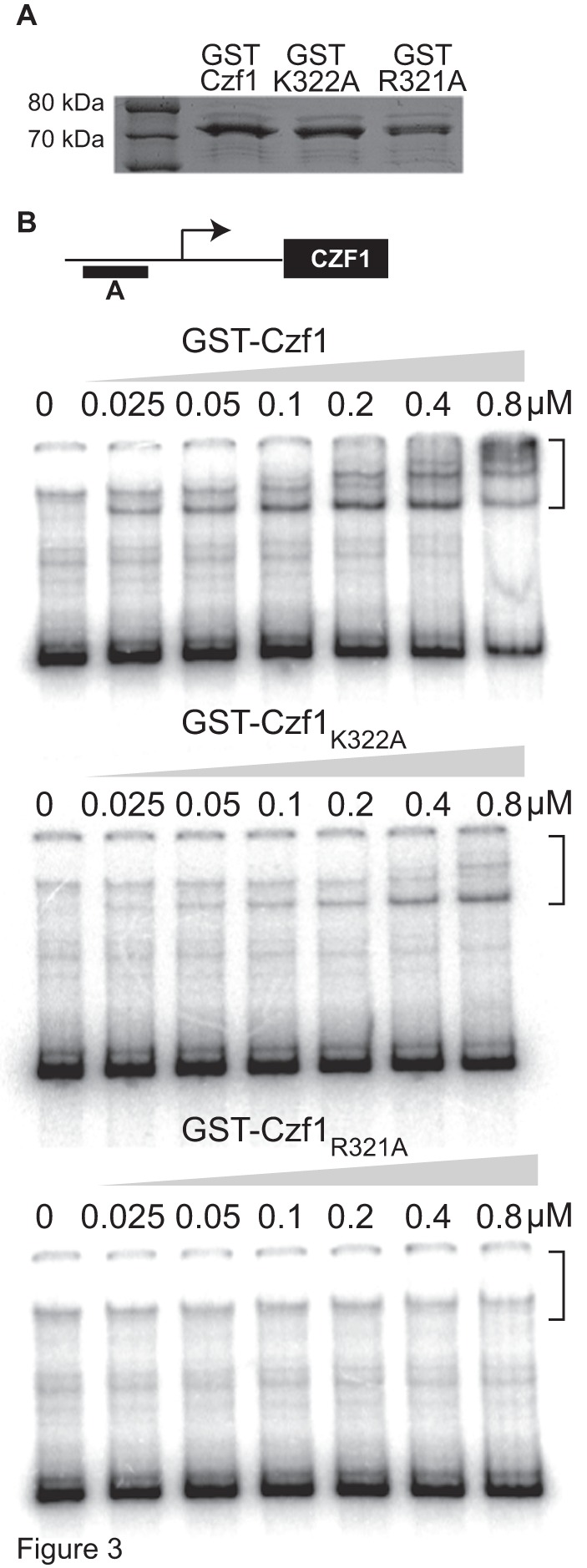
EMSA of wild type and mutant Czf1p binding to the *CZF1* promoter region. (Panel A) SDS PAGE analysis of 500 ng of purified wild type and mutant GST-Czf1 fusion proteins stained with Coomassie Blue. (Panel B) Diagram of the *CZF1* promoter region (not drawn to scale). The DNA fragment used in this study (fragment E, 565 bp) is represented as a black rectangle and is located −3381 to −2816 from the ATG start of the *CZF1* ORF. The transcriptional start site is represented as an arrow and is located at −2065 from the ATG [23]. (Panels C–E) Increasing amounts of GST-Czf1p were incubated with ^32^P end-labelled fragment E. The samples were analyzed by electrophoretic mobility shift assay and phosphorimaging. Brackets indicate shifted fragments. All EMSA experiments were repeated at least 3 times and a representative experiment is shown.

Czf1p binds to specific fragments of the *CZF1* promoter region, but not to promoter sequences of the *ACT1* and *SOD3* genes [23]. To test the DNA-binding activities of the Czf1p_R321A_ and Czf1p_K322A_ mutants, GST fusions of wild-type and mutant Czf1p were expressed in *Escherichia coli* and purified by standard methods (FIG 3A).

To test the abilities of these proteins to bind DNA we used a known Czf1p target DNA sequence (fragment E) located at positions −3381 to −2816 relative to the start of the *CZF1* ORF. This region of the promoter is immediately upstream of the transcriptional start site [23].

Figure 3B shows that DNA-binding by wild-type GST-Czf1p was detectable at a concentration as low as 25 nM and became more robust as protein concentration increased. At lower protein amounts, a single protein-DNA complex was observed, while at higher protein concentrations multiple complexes were detected. In previous studies, we showed that the two complexes formed by binding of Czf1p to fragment E were not observed when another fragment of the *CZF1* promoter was used as the probe, demonstrating binding specificity [23].

Binding of GST-Czf1_K322A_ to DNA was detectable at protein concentrations of 25–50 nM but the amount was low and the lower molecular weight species was most predominant. At 0.8 µM the pattern of protein-DNA complexes was different from that of wild-type GST-Czf1p. Thus, to achieve the level of binding seen with a given concentration of WT GST-Czf1p, higher concentrations of GST-Czf1_K322A_ were required.

Binding of GST-Czf1_R321A_ had a more dramatic defect and no DNA-binding activity was observed at any protein concentration tested. Therefore, one highly conserved residue within the Cys_6_Zn_2_ motif was essential for Czf1p target DNA-binding and important for Czf1p function as a regulator of filamentation.

### Czf1p DNA-binding and interaction with Efg1p are independent of one another

Epistasis studies point to a Czf1p and Efg1p genetic interaction during matrix-embedded growth [14]. Yeast-two-hybrid analysis further supports the notion of an in vivo physical interaction between the two transcriptional regulators. The significance of this physical interaction is unclear.

**Figure 4:**
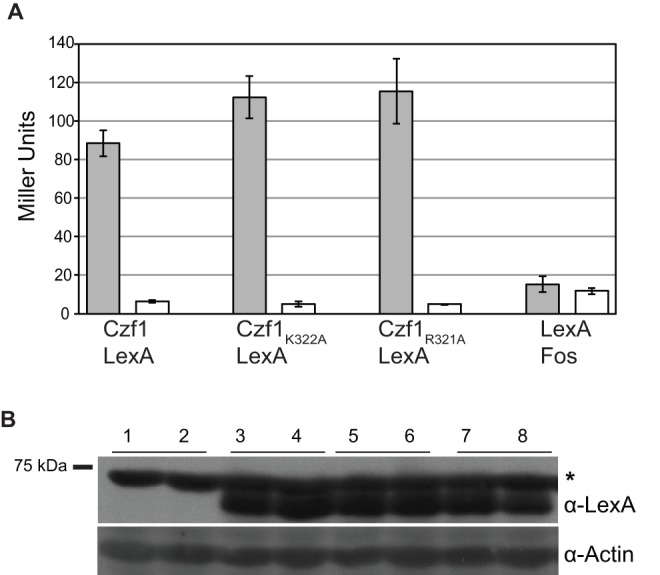
Wild type and mutant Czf1p interact with Efg1p in a yeast two-hybrid assay. *S. cerevisiae* strains carrying a *lacZ* reporter plasmid, a *lexA* DNA-binding domain bait fusion (indicated below columns) and a Gal4 activation domain prey fusion (black bars, Gal4AD-Efg1; white bars, Gal4AD-Slk19) were grown in synthetic complete medium lacking uracil, leucine and histidine (CM-ULH) and β-galactosidase activity was determined using *o*-nitrophenyl β-D-galactopyranoside (ONPG) as the substrate. Analysis was performed in triplicate and mean and standard deviation are shown. Differences in activity of Czf1-LexA fusions with Gal4AD-Efg1p and the same fusion with Gal4AD-Slk19 were statistically significant (p<5×10^−4^ for all Czf1-LexA fusions; two tailed *t* test). (Panel B) *S. cerevisiae* strains were grown in CM-ULH and immunoblot analysis was performed on crude extracts using an anti-LexA antibody to visualize Czf1-lexA protein fusions. Actin was detected as the loading control. Samples from two independent cultures of a given strain are shown. Lanes 1 and 2, strain with *lacZ* reporter and Gal4AD-Efg1 fusions only; lanes 3 and 4, strains carrying *lacZ* reporter, Gal4AD-Efg1 and *CZF1*-lexA fusion; lanes 5 and 6, strains carrying *lacZ* reporter, Gal4AD-Efg1 and *czf1_K322A_*-lexA fusion; lanes 7 and 8, strains carrying *lacZ* reporter, Gal4AD-Efg1 and *czf1_R321A_*-lexA fusion. A cross-reacting band, marked with *, is apparent above the Czf1-LexA fusion.

To test the effects of the *czf1_R321A_* and *czf1_K322A_* mutations on Czf1p-Efg1p two-hybrid interaction, we performed a yeast two-hybrid assay. *S. cerevisiae* strain EGY40 carrying a *lacZ* reporter gene with *lexA* operator sites was transformed with LexA DNA-binding domain plasmids (either *CZF1–lexA* or the control fusion *lexA-FOS*). Gal4 activation domain fusions (either GAL4AD-Efg1 or the control fusion GAL4AD-Slk19) were introduced into these strains. As expected, strains carrying the control fusion proteins, Gal4AD-Slk19 and LexA-Fos produced low levels of β-galactosidase activity (FIG 4A), demonstrating that these fusions did not interact with Czf1p and Efg1p, respectively, nor did they interact with one another. β-galactosidase activity in transformants carrying wild-type Czf1-LexA and Gal4AD-Efg1 was higher than that of transformants carrying control fusions (Gal4AD-Slk19 and LexA-Fos) (FIG 4A), consistent with previous results [14]. Importantly, the Czf1_R321A_ and Czf1_K322A_ fusion proteins interacted with Gal4AD-Efg1 similarly to wild-type Czf1p (FIG 4A). In addition, wild-type and mutant Czf1p fusions showed similar protein levels (FIG 4B).

Therefore, by individually introducing single amino-acid changes into the zinc-finger motif of Czf1p, we generated *czf1* mutants that were defective in DNA-binding, but not in interaction with Efg1p. As a result, we separated the two known activities of Czf1p and showed that a DNA binding defect confers a *czf1* mutant phenotype. These results provide the first evidence of a key role for the DNA-binding activity of Czf1p in regulating the morphological yeast-to-hyphal transition triggered by matrix-embedded growth.

### Interaction between Czf1p and Efg1p has a minor role during matrix-embedded growth

To test the importance of the physical interaction between Efg1p and Czf1p we analyzed a previously characterized mutant form of Efg1p, Efg1-D3, that is compromised in the ability to bind Czf1p in the two-hybrid assay [28]. Efg1-D3 is deleted for amino acids 131–202, adjacent to the APSES domain of the protein [28]. Wild-type *EFG1* and *EFG1-D3* were introduced into an *efg1*Δ strain background and the ability of the strains to produce filamentous colonies during embedded growth at 23°C was measured.

**Figure 5:**
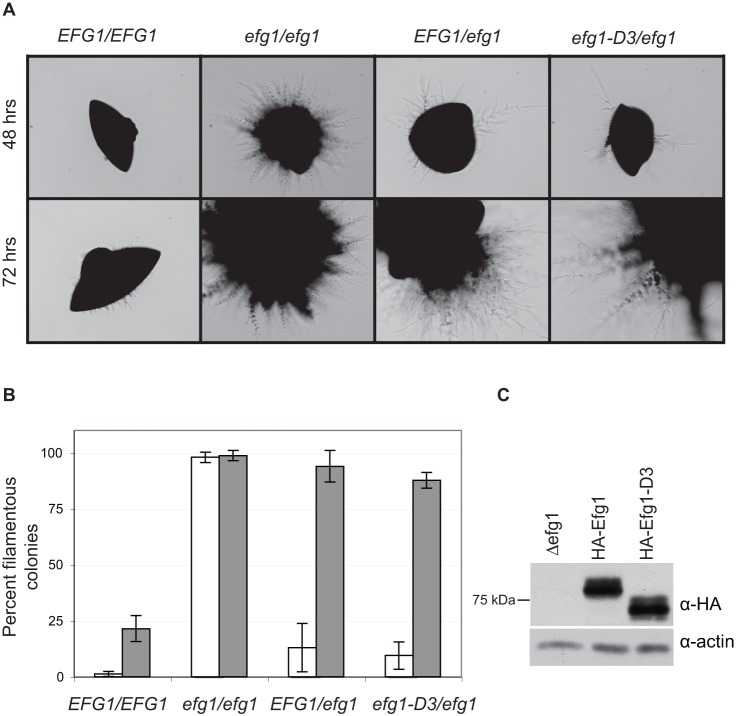
A strain carrying an *efg1* mutant defective in interaction with Czf1p behaves like a strain carrying one allele of wild-type Efg1p during matrix-embedded growth. (Panel A) Strains with the genotypes shown at the top of the figure were grown embedded in YPS 1% agar for 72 hours at 23°C. Two independent isolates of each strain were tested in duplicate. Representative colonies were photographed at 10x at the times indicated. (Panel B) Quantitative representation of the percentage of filamentous colonies at various times. Data show the mean of the 4 determinations and the standard deviation. Black bar, 48 hours; grey bar, 72 hours. Differences in percent filamentous colonies between *efg1*Δ mutant and other strains at 2 days were statistically significant (p<10^−5^; two tailed *t* test). (Panel C) Cells were grown in liquid YPD at 30°C and Efg1p proteins were detected in cell extracts (45 µg total protein) by immunoblotting with anti-HA antibody. Actin was detected with anti-actin antiserum as a loading control.

As shown in Figure 5A and 5B, at a time when the wild-type strain maintained growth in the yeast state, an *efg1*Δ null strain was hyperfilamentous, consistent with previous results [14]. Introducing one copy of *EFG1^+^* partially complemented the *efg1*Δ mutant phenotype, as observed previously. A strain carrying *EFG1-D3* behaved very similarly to the wild-type complemented strain. By detecting the HA tag at the N-terminus of the protein, we showed that the wild-type and mutant Efg1p were produced in similar amounts, consistent with previous reports [28]. Because the *efg1*Δ *cph1*Δ double null mutant behaved similarly to wild-type *C. albicans* [14], *EFG1*-D3 was not tested in this strain background.

In summary, during matrix-embedded growth, the *EFG1-D3* allele had wild type filamentation. Previous results showed that *EFG1-D3* has no defect in hyphal induction in various other media at 37°C, but has a slight defect in the maintenance of the white state in a WO-1 strain of *C. ablicans* [28]. Thus, the interaction between Czf1p and Efg1p mediated by the D3 region of Efg1p is dispensable for the control of morphogenesis during embedded growth, as well as under other conditions.

## Discussion

The contributions of the two known activities of Czf1p to its function as a positive regulator of filamentation had not been previously explored. Here, for the first time, we provide evidence for the biological significance of the DNA-binding activity of Czf1p. A *czf1* mutant, Czf1_R321A_, was defective during *in vitro* DNA-binding using a target sequence from the *CZF1* promoter region. Importantly, this mutant was also defective in the yeast-to-hyphal transition during matrix-embedded growth at 25°C. The importance of Arg321, a highly conserved residue within the Cys_6_Zn_2_ motif of Czf1p, for DNA binding is consistent with the role of the corresponding residue within the same sub-region of other well-studied members of this class of transcriptional regulators [27], [29], [30], [31], [32]. For example, substitution of Glu for Lys at position 17 of *S. cerevisiae* Gal4 (analogous to Arg321 of Czf1p) and substitution of Ala for Lys at position 44 in Leu3 (analogous to Lys322 of Czf1p) lead to an approximately 20-fold and 15-fold reduction in DNA binding activity, respectively [31], [32]. Furthermore, substitution of Lys322 led to reduced DNA binding and a defect in matrix-embedded filamentation. In contrast, mutant Czf1_T328A_ exhibited no defect in filamentation. This result is consistent with previous structural studies of other members of the Cys_6_Zn_2_ family, which showed that residues found in this position and its proximity are facing away from DNA rather than contacting the DNA [29], [30].

Interestingly, the DNA-binding activity of Czf1p was separated from its ability to physically interact with Efg1p. Czf1_K322A_ and Czf1_R321l_ although defective in DNA-binding, were able to interact with Efg1p. Importantly, our data indicate that the Czf1p-Efg1p interaction detected by two-hybrid analysis does not play an essential role in the ability of Czf1p to antagonize Efg1p inhibitory function on filamentation. The *EFG1-D3* mutant allele encoding a mutant Efg1p that is defective in interaction with Czf1p [28], complemented an *efg1*Δ null strain to the same extent as wild type *EFG1*. If the physical binding of Czf1p to Efg1p were the key mechanism to relieving Efg1p-mediated repression of filamentation, the expected phenotype would have been a defect in filamentation under embedded conditions and this phenotype was not observed.

Our findings are consistent with the model that Czf1p exerts its positive influence on filamentation and relief of inhibition by Efg1p, by acting at promoters via its DNA-binding activity. For example, at the *CZF1* promoter region both Czf1p and Efg1p associate with sequences located upstream of the *CZF1* ORF. Importantly, in the cell, Czf1p and Efg1p bind independently to the *CZF1* promoter region [24]. Recent genome-wide association studies conducted by ChIP-chip [21] confirm that Efg1p associates with the *CZF1* promoter region. In addition, in yeast form cells, Efg1p binds to the promoters of several genes encoding negative (Tcc1p, Nrg1p, Rfg1p and Cpp1p) and positive (Tec1p and Def1p) regulators of filamentation [21]. We propose that the effects of Efg1p on these (or other) promoters result in repression of filamentation. In our model, during growth under matrix-embedded conditions, Czf1p, via its DNA-binding activity, acts at these or other promoters resulting in reversal of Efg1p-mediated repression of filamentous growth. It appears that the Czf1p-Efg1p physical interaction is dispensable for this event. This interaction may be required for other functions of Czf1p or Efg1p.

When Czf1p levels are high enough, we propose that Czf1p modulates and shuts down its own expression [23]. Therefore, once its role as a positive regulator has been fulfilled, a negative autoregulatory loop takes place. The transient function of Czf1p in this model is analogous to the transient role of Czf1p in regulation of switching between the more common “white” phenotype and the mating competent “opaque” phenotype [17]. Temporal regulation of multiple factors involved in the control of the yeast-to-hyphal transition is thus a crucial mechanism for regulating the different stages of initiation and maintenance of filamentous growth [11], [21], [33].

The functional relationship between Czf1p and Efg1p in the context of the yeast-to-hyphal transition is apparent only when growth takes place within a semi-solid material at temperatures at or below 35°C. These growth conditions likely simulate a specific physical environment encountered during invasion into tissue in particular parts of the body. In this environment, a distinct Czf1p/Efg1p-dependent genetic program controlling morphogenesis is important. To understand more broadly how morphogenesis is triggered during infection of a human host, it will be of interest to understand the contributions of the many signaling pathways that regulate filamentation in different environments.

## Materials and Methods

### Strains and media

*C. albicans* strains used in this study are listed in Table 2. *C. albicans* cells were routinely grown in YP medium (1% yeast extract, 2% Bacto peptone) with either 2% glucose (YPD) or 2% sucrose (YPS). For embedding experiments, 1% agar was used. For growth of CKY154 [*ura3*Δ *czf1*Δ *cph1*Δ mutant] [13] and its derivatives carrying Myc-tagged *CZF1* alleles, uridine was added to the growth medium at 162 μg/ml. For *czf1* mutant analysis, plasmids encoding Myc-tagged *CZF1* (wild type or mutant) under control of the maltase promoter were linearized by digestion with BsgI and integrated at the *ADE2* locus of strain CKY154. Transformants were selected for Nourseothricin resistance (200 μg/mL) and integrations were confirmed using primers ADE2-For1 and ADE2-Rev2 (Table 3). For *efg1* mutant analysis, plasmids pTD38-HA, pTD38-HA-Efg1 and pTD-HA-Efg1-D3 [28] encoding HA-tagged versions of *EFG1* under control of the endogenous promoter were linearized by digestion with PacI, integrated at the *EFG1* locus of strain Can35 [*efg1*Δ mutant] [5] and correct integrations were confirmed by PCR with primers EFG1-7a and EFG1-7b for vector alone and EFG1-10a and EFG1–10b for *EFG1* containing plasmids. *E. coli* DH5α[34], and GM2163 (*dam^−^dcm^−^*; New England Biolabs) were used to propagate plasmids. *E. coli* strain BL21 [35], [36] was used for expression and purification of recombinant Czf1p. Bacterial cells were cultured in Luria broth or on Luria plates with ampicillin added to a concentration of 100 µg/ml. *S. cerevisiae* strain EGY40 [37] was used for yeast-two-hybrid analysis carrying the bait and prey fusions, and was grown routinely in complete minimal media lacking uracil, leucine and histidine.

**Table 2:** *C. albicans* stains used in this study.

Name	Genotype	Source or reference
	***C. albicans***	
SC5314	Clinical isolate	[41]
CAI-4	SC5314 Δ*ura3*::*imm434*/Δ*ura3*::*imm434*	[41]
CKY154	CAI-4 *czf1::hisG/czf1::hisG cph1::hisG/cph1::hisG*	[13]
Can35	CAI-4 *efg1::hisG/efg1::hisG* Δ*ura3*::*imm434/*Δ*ura3*::*imm434*	[5]
IPC11	CKY154 *ade2::*pDBI52-SAT	This study
IPC22	CKY154 *ade2::*pDBI-Czf1-13xmyc-SAT	This study
IPC31	CKY154 *ade2::*pDBI-Czf1_K322A_-13xmyc-SAT	This study
IPC42	CKY154 *ade2::*pDBI-Czf1_R321A_-13xmyc-SAT	This study
IPC51	CKY154 *ade2::*pDBI-Czf1_T328A_-13xmyc-SAT	This study
IPE1.3	HLC67 *efg1::hisG/efg1::*pTD38	This study
IPE2.3	HLC67 *efg1::hisG/efg1::* pTD38-EFG1	This study
IPE7	HLC67 *efg1::hisG/efg1::*pTD38-EFG1-D3	This study
	***S. cerevisiae***	
EGY40	*MAT ura3*–*1 his3*–*11 trp1*–*1 leu2*–*3,112*	[37]

**Table 3:** Primers used in this study.

A. *czf1* mutant construction by overlap PCR	
R321A–F	CGTATGGGATGTCTTACATGCCGTCAAGCAAAGAAACGTTGTTGTGAAACAAGACCAAGG
R321A–R	CCTTGGTCTTGTTTCACAACAACGTTTCTTTGCTTGACGGC
K322A–F	GGGATGTCTTACATGCCGTCAAAGAGCTAAACGTTGTTGTGAAACAAGACCAAGG
K322A–R	TCTTTGACGGCATGTAAGACATCCC
T328A–F	GCCGTCAAAGAAAGAAACGTTGTTGTGAAGCAAGACCAAGGTGTACTGAGTGCAC
T328A–R	TTCACAACAACGTTTCTTTCTTTGACGGC
B. Construction of myc tagged *CZF1* alleles	
SAT1-F	TGCTCTAGATCATAAAATGTCGAGCGTCAAAACT
SAT1-R	GGTGAATTCGAATTCGAACTTCCTGCAGGACCACCTT
MycF	GGTAGGCCTGGTACCATATACCGGTGGTGGTCGGATCCCCGGGTTAATTAA
MycR	CCTCCAACACAGAGAAGCCTCGAGCGCGAATTCACTAGTGATTGATTAA
3UTR-F	CAATCACTAGTGAATTCGCGCTCGAGGCTTCTCTGTGTTGGAGGGATA
Czf-Stu	GCAGGCCTTATCTGTTCACCCCATTTCTTTCA
Czf-Xba	GCTCTAGAATTTCGTTTTGCTGGTGCTGTG
Czf-Kpn1	GCAGGTACCTATCTGTTCACCCCATTTCTTTCA
Czf-AgeI	GTGACCGGTTTTACTTCTGTATTCAACAATACC
C. Construction of Czf1-lexA fusions:	
BOPO1	GGTGAATTCCATTCAAACGAAAACTATCTGGT
BOPO2	GGTGGATCCTACTTCTGTATTCAACAATACCT
D. Construction of GST-Czf1 fusions	
POM1-F	CGGGATCCATGAGTTCAATACCCAATATCAAT
POM1-R	CGAATTCTTATTTACTTCTGTATTCAACAATAC
E. Amplification of fragment E for mobility shift assay analysis	
Czf1A–F	CAAAACAAGCCAGAATAAAAATA
Czf1A–R	ACCGGAAGTACCAAAAGAGTC
F. Confirmation of proper integrations of CZF1-myc and HA-EFG1 constructs in *C. albicans*:	
ADE2 For1	CGGTACAATCTTGTCAATGAGATGGA
ADE2 Rev2	ATGCTTCCGGCTCGTATGTTGTGT
EFG1-7a	ACTGAGGGCTTTGGTCGTGGT
EFG1-7b	ATCCCTGCAGCCCGGGGAAT
EFG1-10a	ACAGCCAGAGTCAAGCTAAAGCAAG
EFG1-10b	ATGCAGCTCCCGGAGACGGT

### Matrix-embedding assay

Growth and embedding of *C. albicans* cells in agar medium was performed as previously described [13]. Briefly, following 4 hours of growth of a diluted overnight culture, *C. albicans* cells were embedded in YPS containing 1% molten agar and grown at 25°C for *czf1* mutant analysis and 23°C for *efg1* mutant analysis. Uridine was added to 162 µg/ml for embedded growth of Ura^−^ strains. A colony was considered to be filamentous if more than 20 filaments protruding from the edge of the colony were visible. 100 colonies were counted for a given plate. Each assay described was performed at least 3 times.

### Plasmids

Plasmids used in this study are listed in Table 4.

**Table 4:** Plasmids used in this study.

Name		Source or reference
	***C. albicans*** ** plasmids**	
pDBI52		[13]
pTONY1	*MALT*pr-*CZF1* ^+^	Brown and Kumamoto, unpublished
pDBI-SAT	pDBI52, *SAT1*	This study
pIP18	pDBI-Czf1-13xMyc-3UTR-SAT	This study
pIP20	pDBI-Czf1_K322A_-13xMyc-3UTR-SAT	This study
pIP21	pDBI-Czf1_R321A_-13xMyc-3UTR-SAT	This study
pIP22	pDBI-Czf1_T328A_-13xMyc-3UTR-SAT	This study
	pTD38	[28]
	pTD38-EFG1	[28]
	pTD38-EFG1-D3	[28]
	***E. coli*** ** plasmids**	
	pGST-Czf1	[23]
pIP8	pGST-Czf1_K322A_	This study
pIP9	pGST-Czf1_R321A_	This study
	***S. cerevisiae*** ** plasmids**	
pBOM1a	pLexA-Czf1	[14]
pIP6	pLexA-Czf1_K322A_	This study
pIP7	pLexA-Czf1_R321A_	This study
pKLEF4	pGal4AD-Efg1	[14]
pC1.1.2	pGal4AD-Slk19	[14]
	pLexA-Fos	[14]

#### Czf1p-Myc and HA-Efg1p

Wild-type or mutant Czf1-Myc were expressed from the maltase promoter in plasmid pDBI-Czf1-13xMyc-SAT. First, the vector pDBI52-SAT was constructed by cloning *SAT1* into plasmid pDBI52 [13]. Primers SAT1F and SAT1R were used to amplify a fragment from pSFS2 [38] containing the *SAT1* marker. The PCR fragment was subcloned into vector pDBI52. Cloning of CZF1-13xMyc-3UTR into pDBI-SAT was done in two steps. First, an overlap PCR fragment of 13xMyc and the 3′UTR of *CZF1* was generated with pADH34 [39] amplified with primers MycF and MycR to generate the 13xMyc [AB] fragment and pTONY1 (an unpublished *CZF1* ^+^ plasmid isolated as described in [13]) with primers 3UTR-F and CzfXba to generate the 3′UTR [CD] fragment. The fragment was then cloned into pDBI-SAT creating plasmid pDBI-13xMyc-3UTR-SAT. Second, a PCR fragment containing the *CZF1* ORF and ∼ 200bp upstream sequence was generated by primers Czf1KpnI and CzfAge1 and cloned into pDBI-13xMyc-3UTR-SAT. For this PCR, pMV102 [24] served as a template for wild-type *CZF1* and *czf1* mutant alleles were generated by overlap PCR. The resulting final plasmids carrying Myc-tagged Czf1p variants and used for the embedding assay are: pDBI-Czf1_R321A_-13xMyc-SAT, pDBI-Czf1_K322A_-13xMyc-SAT, pDBI-Czf1_T328A_-13xMyc-SAT.

#### Yeast-two hybrid plasmids

A list of yeast-two-hybrid plasmids used in this study is provided in Table 4. To create plexA-Czf1_R321A_ and plexA-Czf1_K322A_ plasmids pDBI-Czf1_R321A_ and pDBI-Czf1_K322A_ served as templates using primers BOPO1 and BOPO2. The *CZF1* ORF-containing PCR fragment was then digested with EcoRI and BamHI and cloned into vector pNlexA [14] digested with the same enzymes.

#### GST fusion proteins for expression in *E. coli*

Wild-type GST-Czf1 was published previously [23]. To create GST fusions of mutant *czf1*, primers POM1F and POM1R were used to amplify *CZF1* alleles using templates pDBI-Czf1_R321A_ and pDBI-Czf1_K322A_. After subsequent digestion with BamHI and EcoRI the PCR fragments were cloned into plasmid pGEX-6P-2 (GE Healthcare Life Sciences) digested with the same enzymes, giving rise to pGST-Czf1_R321A_ and pGST-Czf1_K322A_.

### Western analysis

Strains producing Myc-tagged Czf1p variants under control of the maltase promoter were grown in YPS at 30°C to OD_600_ of 0.8–1. Strains producing HA-tagged Efg1p variants under transcriptional control of the endogenous promoter were grown similarly and as in [28]. Crude cell extracts were prepared by breakage with glass beads, in the presence of protease inhibitors (2 µM PMSF, one tablet Complete mini protease inhibitor cocktail (Roche) per 10 ml lysis buffer). Lysis buffer, RIPA, was: 50 mM TrisCl, pH 7.5, 150 mM NaCl, 0.1% SDS, 0.5% Sodium Deoxycholate, 1% NP40. Total protein was quantified with the BCA assay (Thermo Scientific). 30 µg of total protein for Czf1-Myc and 45 µg of total protein for HA-Efg1 were used. Proteins were separated by 8% sodium dodecyl sulfate-polyacrylamide gel electrophoresis (SDS-PAGE), and immunoblottings were done using mouse anti-Myc (1∶2,000, Upstate) for Czf1-Myc and mouse anti-HA high-affinity antibody (1∶1,000; Roche) for HA-Efg1. For visualization of Czf1-lexA protein fusion in the yeast-two-hybrid assay, cells were grown to exponential phase, harvested, adjusted to 10 OD_600_ units and crude extracts were obtained using M2 lysis buffer pH 7.0 (20 mM HEPES, 150 mM NaCl) and protease inhibitors (Complete mini protease inhibitor cocktail, Roche). Immunoblottings were done using rabbit anti-LexA antibody (1∶1, 5000, Sigma).

### Purification of GST-Czf1

For purification of GST-Czf1p, 1.2 L of cells were grown to OD_600_≤0.8 at 25°C and induced with 0.5 mM IPTG at 30°C for 2.5 hrs. Cells were harvested and resuspended in ice cold 1xPBS (8 mM sodium phosphate, 2 mM potassium phosphate, 140 mM NaCl, 10 mM KCl, ph 7.4) containing a cocktail of protease inhibitors (Roche, EDTA free) and 2 mM PMSF, and passed twice through a French pressure cell at 14,000 psi. The lysate was cleared by ultracentrifugation at 90,000 r.p.m. for 20 min in a Beckman TLA 100.3 rotor. The cleared lysate was loaded onto a pre-equilibrated glutathione Sepharose column. The resin was washed with three times the bed volume with 1xPBS. Protein was first eluted with 50 mM Tris, pH 8.0, 10 mM reduced glutathione and then with 125 mM Tris, pH 8.0, 150 mM NaCl, 50 mM reduced glutathione. Protein from the second elution was used for gel shift analysis.

### Mobility shift assay

Primers CZF1AF/CZF1AR (Table 3) were used to amplify a 565 bp fragment from the *CZF1* promoter, which was denoted the fragment E [23]. DNA probes were gel-purified and labeled with [*γ*-^32^P] ATP using T4 polynucleotide kinase (New England Biolabs) and the manufacturer's protocol. Mobility shift experiments were modified from previously published procedures [23]. Binding reactions were performed in a 20 µL reaction that contained the following components: 12% (v/v) glycerol, 12 mM HEPES-NaOH (pH 7.9), 4 mM Tris-Cl (pH 7.9), 90 mM KCl, 2 μg poly(dI-dC), 5 μg BSA, 0.625 ng ^32^P-labelled oligonucleotide probe, and varying amounts of protein. After incubation for 30 min at 30°C, the binding mixture was separated on 8% polyacrylamide, Tris-Glycine non-denaturing gel (50 mM Tris pH 8.0, 383.6 M Glycine), at room temperature in the absence of chelating agents. Running buffer was 35 mM HEPES, 43 mM Immidazole, pH 7.4. Dried gels were visualized by phosphorimaging.

### Yeast-two-hybrid analysis

The assay was performed as discussed previously [14]. Briefly, *S. cerevisiae* strain EGY40 carrying the *lacZ* reporter plasmid pSH18–34 [37] was transformed with GAL4*-*AD plasmids (pKLEF4 encoding Gal4AD-Efg1, and pC1.1.2 encoding Gal4AD-Slk19) and LexA-DBD plasmids (pBOM1a encoding Czf1-lexA; Czf1_R321A_-lexA, Czf1_K322A_-lexA, and plexA-Fos) using standard methods [40]. For quantitative analyses, strains were grown overnight in synthetic complete medium lacking uracil, histidine, and leucine. Cells were diluted into fresh medium, grown to an OD_600_ of 0.6–0.9, harvested and ß-galactosidase activity was determined using *o*-nitrophenyl ß-D-galactopyranoside as the substrate. Analyses were done in triplicate. Control fusion proteins not expected to interact with Efg1p and Czf1p are lexA-Fos and Gal4AD-Slk19, respectively.
